# Anion effect controlling the selectivity in the zinc-catalysed copolymerisation of CO_2_ and cyclohexene oxide

**DOI:** 10.3762/bjoc.11.7

**Published:** 2015-01-12

**Authors:** Sait Elmas, Muhammad Afzal Subhani, Walter Leitner, Thomas E Müller

**Affiliations:** 1 CAT Catalytic Center, RWTH Aachen University, Worringerweg 2, D-52074 Aachen, Germany. Fax: +49 241 80 22593; Tel.: +49 241 80 28594; 2 Lehrstuhl für Technische Chemie und Petrolchemie, Institut für Technische und Makromolekulare Chemie, RWTH Aachen University, Worringerweg 1, D-52074 Aachen, Germany

**Keywords:** anion effect, carbon dioxide, CO_2_ chemistry, copolymerisation, polyethercarbonate, zinc catalyst

## Abstract

The choice of the anion has a surprisingly strong effect on the incorporation of CO_2_ into the polymer obtained during the zinc-catalysed copolymerisation of CO_2_ and cyclohexene oxide. The product span ranges from polyethercarbonates, where short polyether sequences alternate with carbonate linkages, to polycarbonates with a strictly alternating sequence of the repeating units. Herein, we report on the influence of the coordination ability of the anion on the selectivity and kinetics of the copolymerisation reaction.

## Introduction

**Scheme 1:**
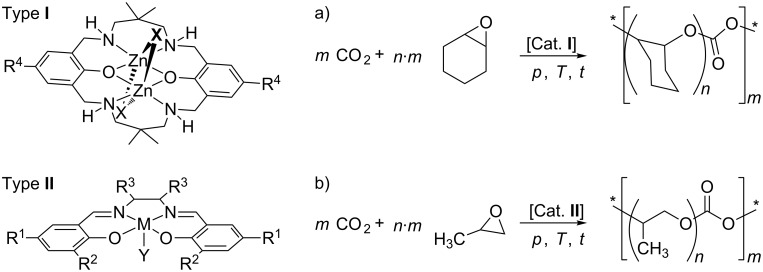
Structural motif of two important types of catalysts and typical substrate specificity in the copolymerisation of CO_2_ and epoxides (*: end groups). Type I: binuclear complexes with a macrocyclic Robson-type ligand framework; Type II: mononuclear complexes with a Salen ligand.

The fixation of carbon dioxide (CO_2_) into polymers [1–3] provides highly promising options for the utilization of CO_2_ [4–7]. The copolymerisation of CO_2_ with epoxides (Scheme 1) is a prime example of a particularly attractive transformation of CO_2_ [8–9] and is at the verge of commercialisation [8]. In this transformation, the low energy level of the CO_2_ molecule is overcome by reacting CO_2_ with an epoxide as energy-rich comonomer [10]. Homogeneous and heterogeneous catalysts are known to catalyse the copolymerisation reaction [5,11–13]. One lead structure (Scheme 1) for catalysing the reaction is based on binuclear complexes with a macrocyclic ligand framework (Type I) [14–16]. The macrocyclic ligand L is a 22-membered Robson-type ligand with four secondary amino and two phenoxy donor groups [17]. The binuclear Zn(II) complex ([LZn_2_X_2_], X = acetate) is substrate specific for the copolymerisation of CO_2_ and cyclohexene oxide [16]. Another established common structural motif is based on Salen-type ligands with a central Co(III) [18–20] or Cr(III) [21–24] atom (Type II). Most catalysts based on Salen-type ligands are substrate specific and are most efficient for the copolymerisation of CO_2_ with propylene oxide (PO). The use of zinc-based catalysts of Type I appears more favourable from an environmental perspective compared to the use of the transition metal cations (Co, Cr) often employed in Type II catalysts, albeit zinc catalysts have lower activity in the copolymerisation of CO_2_ and epoxides [16].

The use of catalysts of Types I and II in the copolymerisation of CO_2_ and epoxides leads commonly to fully alternating polycarbonates. The polymer backbone of such alternating polycarbonates is relatively stiff due to the restricted rotational freedom in the C–O bonds of the carbonate group. For many applications it would be desirable to have a higher – and adjustable – flexibility of the polymer chain as the latter controls many of the physicochemical properties of the polymer, such as the glass-transition temperature (*T*_g_). With incorporated ether linkages, a molecular weight in the oligomer range and at least two terminal OH groups, polyethercarbonates are interesting polyol building blocks in polyurethane chemistry [8].

To keep the catalyst loading during the synthesis of such polyols low, chain transfer between the growing polymer chain and free alcohol groups needs to be realised. In this study, we have addressed such immortal copolymerisation of CO_2_ and epoxides with zinc catalysts. In the Type I lead catalyst [LZn_2_(OAc)_2_] [15], the two acetate counter ions may also act as a starter initiating the polymerisation reaction giving rise to polycarbonates with an acetate end group. Furthermore, these anions strongly coordinate in a bridging fashion to the zinc centre. In consequence, the Lewis acidic zinc centre is initially not accessible for coordination of the substrate giving rise to a certain inhibiting effect. So far, the role of the anion and its effect on the activity and selectivity of the zinc catalysts in the copolymerisation of CO_2_ and epoxides has not yet been fully understood. To study and unravel the role of the anion, we have replaced the two acetate counter anions in the complex [LZn_2_(OAc)_2_] by essentially non-coordinating trifluoromethyl sulphonate (CF_3_SO_3_^−^) or weakly coordinating *p*-toluenesulphonate anions (*p*-TSO_3_^−^). Herein, we report on the effect of the choice of the counter anion on the product selectivity and the activity of the complexes in catalysing the reaction of CO_2_ with cyclohexene oxide.

## Results and Discussion

### Synthesis and characterisation of binuclear [LZn_2_](X)_2_ complexes

**Scheme 2:**
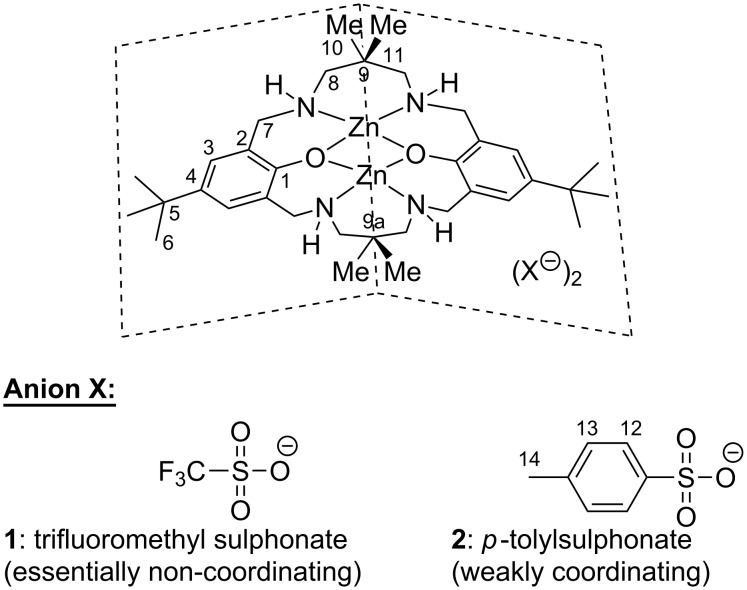
Binuclear Zn(II) complexes [LZn_2_](CF_3_SO_3_)_2_ (**1**, KOP113) and [LZn_2_](*p*-TSO_3_)_2_ (**2**, KOP115) explored in this study (the numbers refer to the assignment of the NMR signals, see also Table 1).

Complexes [LZn_2_]](CF_3_SO_3_)_2_ (**1**) and [LZn_2_](*p*-TSO_3_)_2_ (**2**) (Scheme 2) were prepared by reacting the corresponding zinc salt with the deprotonated macrocyclic ligand H_2_L. Successful complexation was confirmed by the high-field shift of the ^13^C and ^1^H NMR resonances assigned to the aromatic groups of **1** and **2** relative to those of the free ligand. Inspection of the ^13^C APT NMR spectra showed that the position of the signal of the aromatic carbon 2 (for assignment, refer to Scheme 2) was slightly shifted to higher field for **1** (122.0 ppm) relative to **2** (123.0 ppm), suggesting an increased shielding due to lower electron density in the aromatic ring of **1**. This is consistent with the weaker coordinating character of the trifluoromethylsulphonate anion (essentially non-coordinating) in comparison to the *p*-toluenesulphonate anion (weakly coordinating). This interpretation is supported by the IR spectra, where the position of one of the two characteristic S=O stretch vibration bands was significantly red-shifted for **1** (1005 cm^−1^) relative to **2** (1040 cm^−1^), while the position of the second S=O stretch vibration band remained essentially unchanged (**1**: 1190 cm^−1^, **2**: 1180 cm^−1^).

A closer inspection of the ^13^C NMR spectra revealed two clearly separated signals for the methyl groups 10 and 11 (**1**: 28.2 and 20.8 ppm, **2**: 28.3 and 21.2 ppm, respectively), while only one signal at 25.2 ppm was observed for the methyl groups 10 and 11 in the free ligand H_2_L. Also in the ^1^H NMR spectra, the position of the signals assigned to the two methyl groups 10 and 11 was distinctly different (**1**: 0.9 and 1.2 ppm; **2**: 0.8 and 1.1 ppm). Similarly, two separate signals were observed for the methylene protons 8 in the ^1^H NMR spectra of **1** (2.6 and 3.1 ppm) and **2** (2.4 and 2.5 ppm), while only one signal was observed for the methylene group in the ^13^C NMR spectra. Such an NMR pattern could arise when the ligand in **1** and **2** adopts a roof-shaped geometry, as indicated in Scheme 2. An alternative explanation may be anisotropic effects induced by the sulphonate anion through formation of a tightly associated ion pair in the relatively non-polar solvent CDCl_3_.

**Table 1:** Position and assignment of the NMR signals of complexes **1** and **2** in comparison to the parent macrocyclic ligand H_2_L.

Assignment^a^	[L_2_Zn](CF_3_SO_3_)_2_ (**1**)		[L_2_Zn](p-TSO_3_)_2_ (**2**)		LH_2_	
	^1^H	^13^C	^1^H	^13^C	^1^H	^13^C

1	–	n.o.^b^	–	n.o.^b^	–	154.6
2	–	122.0	–	123.0	–	124.2
3	6.88	128.0	6.80 or 7.67	127.7	6.94	125.0
4	–	n.o.^b^	–	n.o.^b^	–	140.8
5/9	–	33.5 33.6	–	33.5 33.7	–	33.9 34.6
6	1.25	31.5	1.28	31.8	1.26	31.6
7	4.30	55.9	4.08	55.8	3.74	53.3
8	3.10 2.62	63.2	2.44	63.0	2.52	59.8
10/11	1.18 0.97	28.2 20.8	1.02 0.89	28.3 21.2 or 21.3	1.01	25.2
12/13	–	–	7.67 or 6.80	128.3	–	–
14	–	–	2.19	21.3 or 21.2	–	–
-OH/-NH	2.86	–	2.84	–	n.o.^b^	–

^a^For the numbering refer to Scheme 2; ^b^n.o. not observed.

For **1** and **2**, mass spectrometry revealed characteristic series of salt-like agglomerates [LZn_2_]X, [LZn_2_]_2_X_3_, [LZn_2_]_3_X_5_ and [LZn_2_]X_3_, [LZn_2_]_2_X_5_, [LZn_2_]_3_X_7_ consistent with weak coordination of the anion to the metal centre. For **1**, an additional signal assigned to the parent divalent cation [LZn_2_]^2+^ was observed, whereas the signal was absent for **2**, which is consistent with the slightly more coordinating nature of the *p*-toluenesulphonate anion. In comparison, the acetate reference complex gave rise to a prevailing signal corresponding to the mass of the non-dissociated complex [LZn_2_(OAc)_2_] [15] which is in full agreement with the strongly coordinating nature of the acetate anion.

### Copolymerisation of CO_2_ and cyclohexene oxide with binuclear [LZn_2_](X)_2_ complexes

**Scheme 3:**
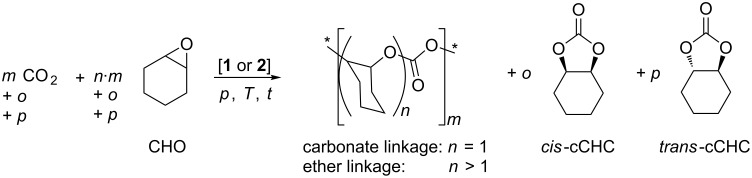
Copolymerisation of CO_2_ and cyclohexene oxide (*: end groups of the polymer chain).

Complexes **1** and **2** were then evaluated as catalysts in the copolymerisation of CO_2_ and cyclohexene oxide CHO (Scheme 3). To obtain insight into the kinetics, the progress of the reactions was monitored with in situ IR spectroscopy and the results are collected in Table 2 (entries 1–4).

Under the applied conditions (100 °C, 20 bar), both **1** and **2** afforded high conversion of CHO. A polymer with the expected molecular weight in the oligomer range and a narrow molecular weight distribution was obtained (Table 2, entries 1 and 2). Closer analysis of the polymer obtained with catalyst **1** revealed that a polyethercarbonate characterised by surprisingly long polyether segments interspaced by carbonate groups was obtained (ratio of carbonate to ether moieties *m*/*n* 4.5/95.5, Table 2, entry 1). This is in agreement with a relatively small consumption of CO_2_ during the reaction. The polymer was obtained in excellent selectivity and only traces of cyclic cyclohexene carbonate (cCHC) were formed as byproduct ((*o*+*p*)/*m* <0.01).

**Table 2:** Yield and selectivity in the copolymerisation of CO_2_ and CHO using complexes **1** and **2** as catalysts and analytical data for the polymers obtained.

Entry	Catalyst	Alcohol (equiv)	Yield^a^ (%)	Selectivity^b^		*M*_n_ (g/mol)	PDI
				(*o*+*p*)/*m*	*m*/*n*		

1	**1**	–	94	<0.01	4.5/95.5	3082	1.64
2	**2**	–	74	0.26^c^	>99.0/1.0	2735	1.33
3	**1**	0.01^d^	72	<0.01	6.6/93.4	3567	1.75
4	**2**	0.01^d^	55	0.02	>99.0/1.0	2019	1.24
5^e^	[LZn_2_(OAc)_2_]	–	59	>0.08	>99.0/1.0	–	–
6 [15]	[LZn_2_(OAc)_2_]	–	55	<0.11	~100/0	–	–

^a^Yield of polymer; ^b^(*o*+*p*)/*m*: ratio of cyclic cyclohexene carbonate to carbonate linkages in the polymer; *m*/*n*: ratio of carbonate to ether linkages in the polymer; ^c^high selectivity to polycarbonate (low value for (*o*+*p*)/*m*) during the initial phase of the reaction, see Figure 2; ^d^Molar ratio of α,ω-dihydroxypolypropylene oxide to CHO 1/72, corresponding to 1 OH group per 36 CHO molecules; ^e^reaction at 90 °C.

**Figure 1:**
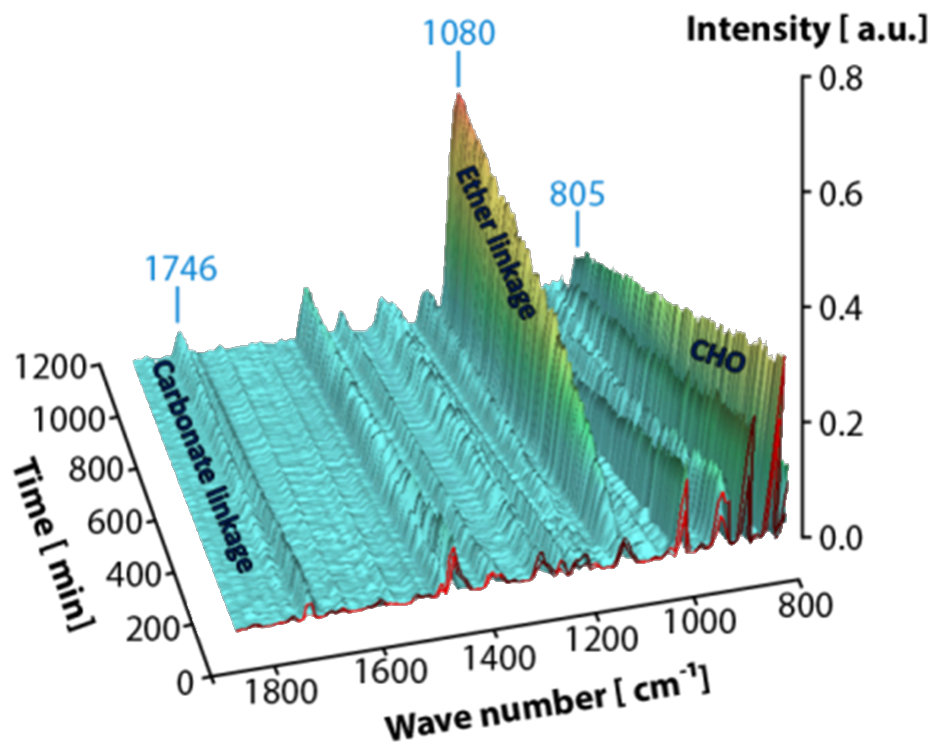
Time-resolved IR spectra of the copolymerisation of CO_2_ and CHO with catalyst **1** showing the formation of carbonate and ether groups in the polymer.

**Figure 2:**
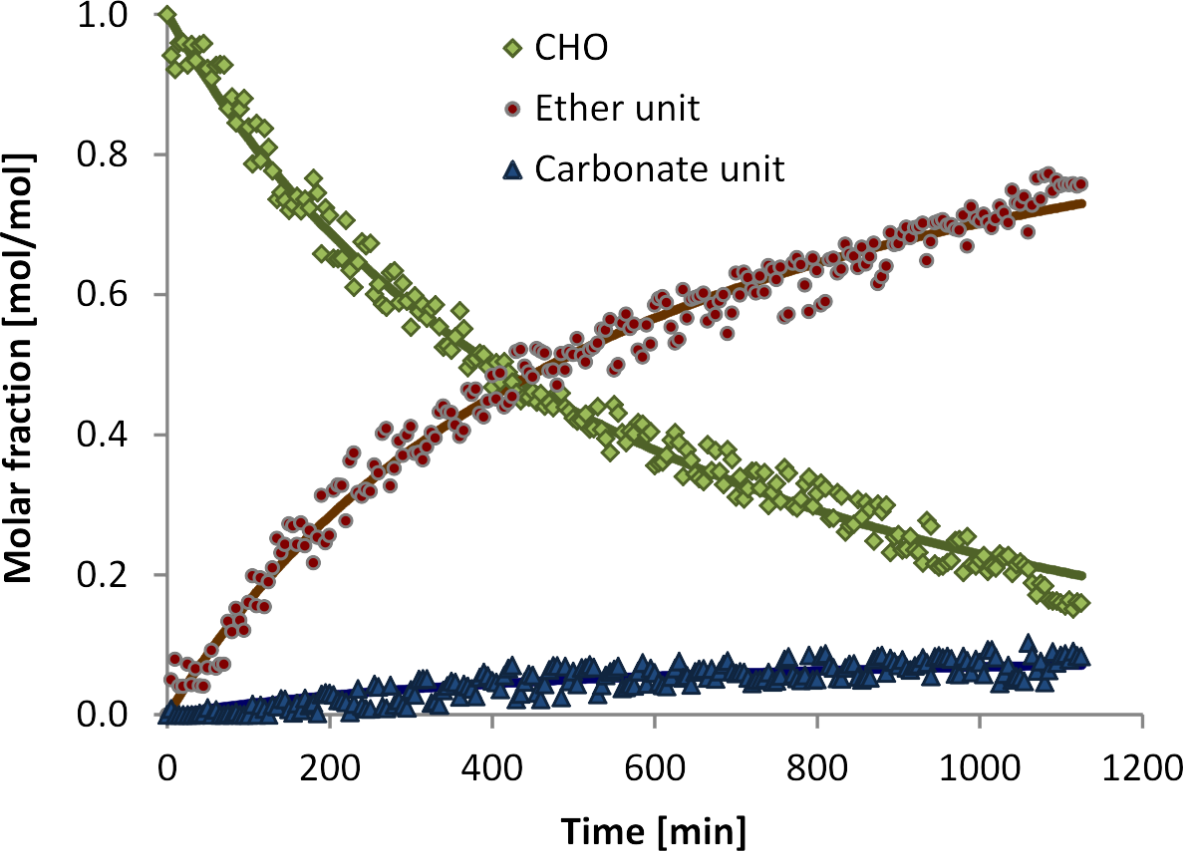
Time–concentration profile of the copolymerisation of CO_2_ and CHO in the presence of catalytic amounts of complex **1** and fit according to a first order kinetic in CO_2_ and in epoxide.

Analysis of the in-situ IR spectra recorded during the reaction revealed the evolution of an intensive band at 1080 cm^−1^ characteristic for the C–O–C bending vibration assigned to the ether linkages, while the intensity of the signal at 805 cm^−1^ characteristic for CHO decreased in parallel (Figure 1). At 1746 cm^−1^, the typical carbonate band [*ν*_st_(C=O)] [25] appeared with low intensity. Quantitative analysis of the time-resolved IR spectra (Figure 2) revealed a profile of epoxide consumption consistent with a first order reaction in both CO_2_ and epoxide. The initial rate for the consumption of CHO was 5.03 mol_CHO_^.^(mol_cat_^.^h)^−1^. Ether and carbonate linkages in the polyethercarbonate product were formed in parallel in a ratio of 9.8. The catalyst was still active after 19 hours reaction time and higher yields can be achieved at prolonged reaction times.

In contrast, with complex **2**, a fully alternating polycarbonate with a high fraction of carbonate linkages was obtained (*m*/*n* >99.0/1.0, Table 2, entry 2). A considerable pressure drop was observed during the reaction consistent with a consumption of CO_2_ in a stoichiometric ratio to CHO. Considerable amounts of cCHC were found as byproduct at the end of the reaction ((*o*+*p*)/*m* 0.26, vide supra). This chemoselectivity was similar to [LZn_2_(OAc)_2_], where a mixture of alternating polycarbonate (*m*/*n* >99.0/1.0) and cCHC was obtained ((*o*+*p*)/*m* <0.08, Table 2, entries 5 and 6).

The results clearly show that the selectivity with respect to the obtained incorporation of CO_2_ into the polymer chain is reversed for complexes **1** and **2**. This is particularly surprising due to the similarity of the sulphonate counter anions and suggests that CO_2_ incorporation is related to subtle differences between the two catalysts. Most likely the differences in coordination strength of the anion to the zinc centre account for this change in selectivity.

**Figure 3:**
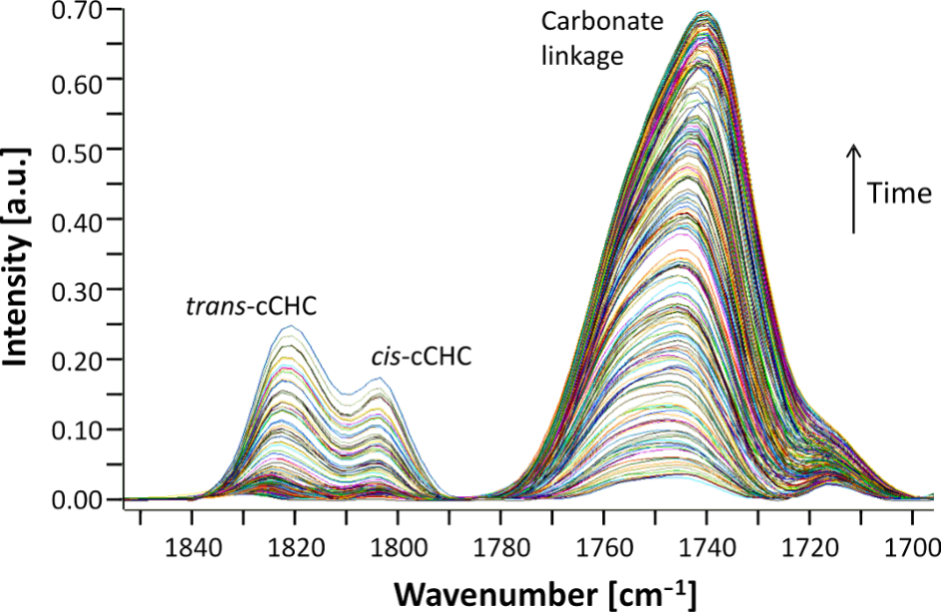
Carbonate region of the time-resolved IR spectra recorded during the copolymerisation of CO_2_ and cyclohexene with catalyst **2**.

**Figure 4:**
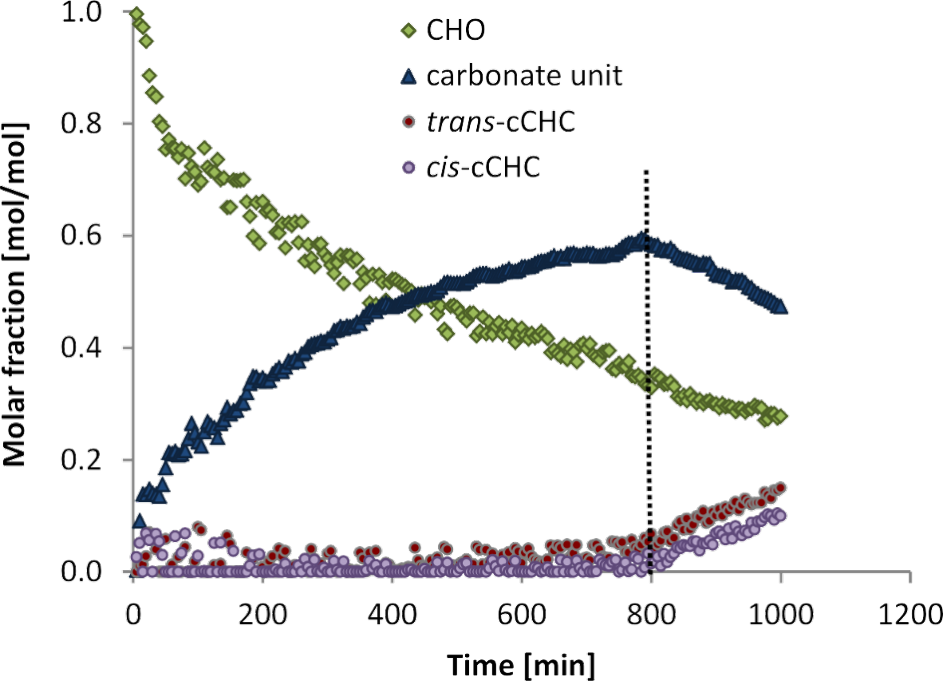
Time–concentration profile of the copolymerisation of CO_2_ and CHO in the presence of catalytic amounts of complex **2**.

The time-resolved IR spectra recorded during the reaction using catalyst **2** indicate a very different regime compared to the reaction with catalyst **1**. During the initial period CHO was consumed with a rate of 13.7 mol_CHO_^.^(mol_cat_^.^h)^−1^. In parallel, two bands with high intensity appeared at 1746 cm^−1^ and 1225 cm^−1^ (Figure 3), which are typical for the [*ν*_st_(C=O)] and [*ν*(C–O)] vibration of polycarbonates, respectively [25]. After 800 min of reaction time, two further carbonate bands assigned to *cis-* and *trans-*cCHC commenced to develop at 1820 cm^−1^ and 1803 cm^−1^, respectively. In parallel, the concentration of polycarbonate decreased (Figure 4). This is consistent with back-biting of free polymer chains, which might be induced by the increasing polarity of the reaction medium leading to an enhanced probability that the polymer chains detach from the zinc centres.

To explore the possibility of an immortal polymerisation [26–27], the reactions were repeated in the presence of an alcohol (α,ω-dihydroxypolypropylene oxide, 1 OH group per 36 CHO molecules). Catalyst **1** afforded essentially the same homopolymerisation product (Table 2, entry 3) albeit in a slightly lower yield (72%). Also, the reaction profile was nearly identical. Analogously, a very similar product was obtained with catalyst **2** when the reaction was performed in the presence of the alcohol (55% yield in polymer, Table 2, entry 4).

**Scheme 4:**
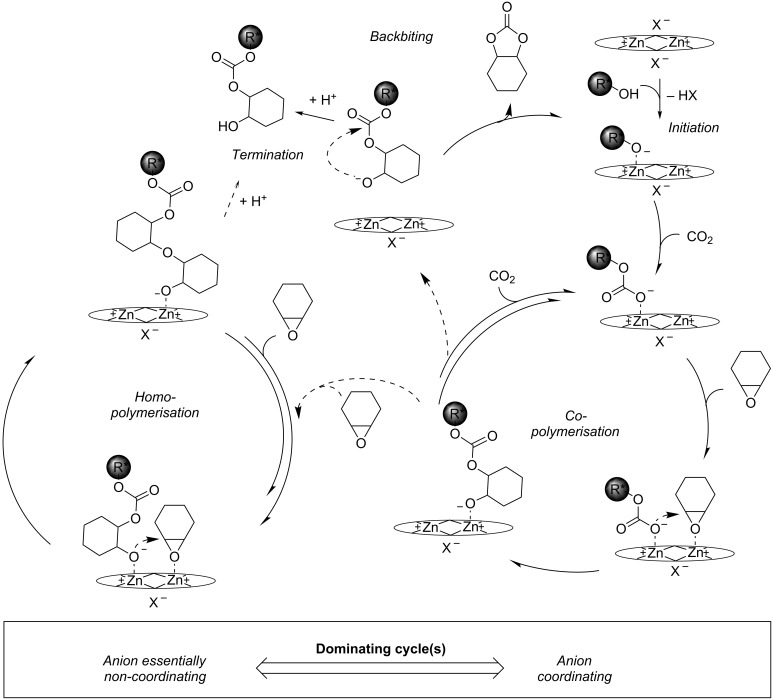
Proposed inner-sphere mechanism for the copolymerisation of CO_2_ and CHO with binuclear zinc complexes (**1**: X = CF_3_SO_3_, **2**: X = *p*-TSO_3_).

These observations are readily explained by a similar mechanism as described for the heterogeneous Zn[Co(CN)_6_] double metal cyanide (DMC) catalyst [8,28], for which an active site comprising two Lewis acidic zinc centres in vicinity had been proposed [29]. Three catalytic cycles, copolymerisation, homopolymerisation and formation of cyclic carbonate are closely linked (Scheme 4) [28]. The reaction is initiated by coordination of an alcoholate to one of the zinc centres. Insertion of CO_2_ into the metal–alcoholate bond provides a coordinated carbonate species [15]. An epoxide molecule coordinates to a neighbouring zinc centre and the nucleophilic attack by the neighbouring carbonate species leads to chain growth. Insertion of the next CO_2_ molecule into the zinc–alcoholate bond closes the copolymerisation cycle. The latter competes with coordination of another epoxide molecule at the zinc centre next to the zinc–alcoholate. Nucleophilic attack of the alcoholate species and coordination of another epoxide molecule on the neighbouring zinc centre closes the homopolymerisation cycle. The cyclic carbonate is formed by backbiting of a terminal alcoholate, when the chain becomes released after preceding insertion of CO_2_. Chains, which dissociate from the surface of the catalyst, restart a catalytic cycle when they re-attach to a free zinc site or react with a coordinated epoxide molecule, while protonation terminates the growth of this particular chain.

In such an inner-sphere mechanism the epoxide molecules compete with the anion in coordination to the zinc centres. In particular, coordination of the epoxide to a zinc centre neighbouring a zinc–carbonate or –alcoholate becomes less likely as coordinating anions are present in the reaction mixture. This readily explains the differences between the CF_3_SO_3_^−^ and the *p*-TSO_3_^−^ complex. In the presence of a CF_3_SO_3_^−^ anion (essentially non-coordinating) coordination of the epoxide to a neighbouring zinc centre is facile. In consequence, the homopolymerisation cycle prevails and mainly polyether segments interspersed with carbonate linkages are formed. In the presence of the *p*-TSO_3_^−^ anion (weakly coordinating), the lower concentration of epoxide molecules coordinating to a neighbouring zinc centre leads to an increase in the probability that a CO_2_ molecule is inserted into the zinc–alcoholate bond. In consequence, the copolymerisation cycle dominates leading with high selectivity to the alternating polycarbonate. In the presence of the OAc^−^ anion, a similar pathway as for the *p*-TSO_3_^−^ anion may be followed or an outer-sphere mechanism with external attack of a chain dissociated from the surface of the catalyst on a coordinated epoxide molecule may be present [5]. The propensity to dissociation of the polymer chain depends strongly on the polarity of the reaction medium. The probability that the growing polymer chain detaches increases with the polarity of the medium leading to backbiting as observed with catalyst **2** at higher conversions.

An alternative model may involve a parallel cationic polymerisation mechanism of cyclohexene oxide with the more Lewis acidic complex **1**, which competes with the regular insertion mechanism depicted above. In contrast to CF_3_SO_3_^−^, the *p*-TSO_3_^−^ anion may be sufficiently nucleophilic to open an epoxide molecule coordinated to the Zn cation, consequently initiating the growth of the polymer chain in an analogous manner to acetate. Conversely, the less nucleophilic CF_3_SO_3_^−^ cannot open a coordinated epoxide molecule and a cationic homopolymerisation of cyclohexene oxide becomes the prevailing chain-growth mechanism. It is important to note that in a cationic mechanism, CO_2_ molecules cannot be inserted as easily, hence, leading to the incorporation of negligible amounts of CO_2_.

Non-coordinating anions lead to a more electrophilic zinc centre with a stronger Lewis acidity, thereby triggering the homopolymerization in case of the CF_3_SO_3_^−^ anion. In this context, it is known that the rate of the CO_2_ insertion into metal–oxygen bonds depends critically on the nucleophilicity of the metal centre [26]. Nevertheless, it is surprising how sharply the selectivity of the CO_2_/epoxide coupling reaction reverses upon a slight change in the anion.

## Conclusion

In summary, the nature of the anion has a striking effect in the copolymerisation of CO_2_ and cyclohexene oxide with binuclear zinc catalysts of Type I. The proposed mechanistic model readily explains the outstanding selectivities observed with complexes [LZn_2_]X_2_ (**1**: X = CF_3_SO_3_, **2**: X = *p*-TSO_3_) to the polymeric product. With **1**, the formation of polyethercarbonates is preferred, whereas with **2** and the reference catalyst [LZn_2_(OAc)_2_], polycarbonates with a strictly alternating sequence of the repeating units are obtained.

## Supporting Information

File 1 Experimental.
