# Supplementary material for: Anion effect controlling the selectivity in the zinc-catalysed copolymerisation of CO2 and cyclohexene oxide
Source: Beilstein J Org Chem. 2015 Jan 12;11:42–9. doi: 10.3762/bjoc.11.7 (PMC4311667; doi:10.3762/bjoc.11.7)
Supplement: File 1 — Experimental. [file Beilstein_J_Org_Chem-11-42-s001.pdf]

Supporting Information

for

Anion effect controlling the selectivity in the zinc-catalysed copolymerisation of CO_2 and cyclohexene oxide

Sait Elmas[1], Muhammad Afzal Subhani[1], Walter Leitner[2] and Thomas E. Müller[1,*]

Address: [1]CAT Catalytic Center, RWTH Aachen University, Worringerweg 2, D-52074 Aachen, Germany. Fax: +49 241 80 22593; Tel.: +49 241 80 28594 and [2]Lehrstuhl für Technische Chemie und Petrolchemie, Institut für Technische und Makromolekulare Chemie, RWTH Aachen University, Worringerweg 1, D-52074 Aachen, Germany

Email: Thomas E. Müller - Thomas.Mueller@CatalyticCenter.RWTH-Aachen.de

*Corresponding author

Experimental

General procedures and materials

All chemicals were obtained from commercial suppliers and used as received. Cyclohexene oxide (99.7 %) was purchased from Aldrich, dried by distillation from CaH_2 and stored over molecular sieves. The water content was determined to 20.1 ppm by Karl-Fischer-titration. Carbon dioxide (99.995%) was purchased from Westfalen AG. Air- and water-sensitive compounds were handled in an atmosphere of dry argon using standard Schlenk techniques.

IR spectra were recorded on a Bruker MATRIX-MF spectrometer equipped with a 3.17 mm high-pressure ATR-IR fibre optical probe (90° diamond prism with 1 × 2 mm basal area and 1 mm height as ATR element, 2 × 45° reflection of the IR beam, IR beam coupled *via* fibre optics). The ATR-IR fibre optical probe was fitted into the reactor in such a way that the diamond at the end of the optical probe was immersed entirely into the reaction mixture. IR spectra (average of 100 scans) were recorded time-resolved in the region 4000-400 cm^{-1} with a resolution of 4 cm^{-1} against argon as background and analysed with the software PEAXACT. Initial rates were calculated by fitting the time-concentration profiles with the Runge Kutta method.

The ^{1}H-NMR and ^{13}C-NMR spectra were recorded at 400 MHz and 125 MHz, respectively, using a Bruker AV400 spectrometer. Chemical shifts are reported relative to tetramethylsilane (TMS).

Molecular weights were determined by gel permeation chromatography on a SECurity GPC System from PSS Polymer Service, which was equipped with two columns (PSS SDV linear M, 8×300 mm, 5 µm) and RID detector. The flow rate was 1.0 ml/min. The procedure was in accordance with DIN 55672-1: "Gel permeation chromatography, Part 1 - Tetrahydrofuran as the eluting agent". Polystyrene samples of known molecular weight were used for calibration.

Synthesis of $[H_4L](ClO_4)_2$

4-*tert*-Butyl-2,6-diformylphenol (2.68 g, 13.0 mmol), sodium perchlorate (6.27 g, 51.2 mmol) and acetic acid (1.47 mL, 25.7 mmol) were dissolved in methanol (180 mL). The solution was stirred and heated to 70 °C. When the mixture started to reflux, 2,2-dimethyl-1,3-propanediamine (1.56 mL, 13.0 mmol) in methanol (60 mL) was added dropwise. Thereafter, the heating was switched off and the mixture stirred overnight at room temperature. All volatiles were removed *in vacuo* affording to a

bright orange solid. The solid was filtered off, washed three times with cold ethanol and diethyl ether (-78 °C) and dried to give a bright orange product (9.22 g, 11.7 mmol, 90 % yield).

$C_{34}H_{50}Cl_2N_4O_{10}$ (745.69 g/mol); ^{1}H NMR (400 MHz, DMSO d_6) δ/ppm = 13.60 (4H, br s, NH/OH), 8.66 (4H, d, N=CH), 7.64 (4H, s, Ar-H), 3.86 (8H, s, CH_2), 1.27 (12H, s, CH_3), 1.13 (18H, s, CH_3); ^{13}C APT NMR (125 MHz, DMSO d_6) δ/ppm = 176.5, 169.3, 142.5, 136.2, 116.6, 56.0, 45.8, 34.8. Comparison of the NMR spectra with reported values confirms the chemical structure of the product as $[H_4L](ClO_4)_2$ [1,2].

Synthesis of H_2L

A suspension of $[H_4L](ClO_4)_2$ (4.36 g, 5.85 mmol) in methanol (360 mL) was cooled to 0°C. $NaBH_4$ (6.44 g, 170 mmol) in methanol (76 mL) was added slowly. When the orange suspension turned into a clear solution, water was added until a white precipitate started to form. The suspension was then left standing overnight. The white solid was filtered off and the product dried *in vacuo*.

$C_{34}H_{56}N_4O_2$ (552.83 g/mol); ^{1}H NMR (400 MHz, DMSO d_6) δ/ppm = 6.94 (4 H, s, Ar-H), 3.74 (8H, s, CH_2), 2.52 (8H, s, CH_2), 1.26 (18H, s, CH_3), 1.01 (12H, CH_3); ^{13}C APT NMR (125 MHz, DMSO d_6) δ/ppm = 154.6, 140.6, 124.8, 124.0, 59.9, 53.2, 34.9, 33.7, 31.6, 25.2. Elemental analysis: calculated C 73.87, H 10.21, N 10.13, found C 73.82, H 10.22, N 10.07. FTMS + p ESI (m_z, g/mol): found 553.45 (M + 1), 452.33 (M + 1 – 102.12), 351.23 (M +1 – 202.22), 277.28 (M +1 – 176.17), 207.14 (M +1 – 346.31), 175.11 (M +1 – 378.34); FTMS – p ESI (m_z, g/mol): found 551.43 (M – 1), 375.31 (M – 1 – 176.11) and 275.21 (M – 1 – 276.22). Comparison of the NMR spectra with reported values confirms the chemical structure of the product as H_2L [1,2].

Synthesis of $[LZn_2](CF_3SO_3)_2$ (1)

The ligand H_2L (402 mg, 0.725 mmol) was dissolved in dry THF (10 mL) and cooled to 0 °C. NaH (65 mg, 2.708 mmol) was added and the solution allowed to warm slowly to room temperature. After stirring the mixture for 1 hour, zinc (II) *bis*-(trifluoromethyl sulphonate) (0.542 g, 1.49 mmol) was added to the solution. The mixture was stirred overnight. The precipitate was filtered off, washed with THF, dried and extracted with CH_2Cl_2. The CH_2Cl_2 phase was filtered, the CH_2Cl_2 removed in a partial vacuum to yield the product.

$C_{37}H_{56}Cl_2F_6N_4O_8S_2Zn_2$ (1061.14 g/mol); ^{1}H NMR (400 MHz, $CDCl_3$) δ/ppm = 6.88 (4H, s, Ar-H), 3.10 (4H, br d, CH_2), 2.86 (8H, br m, CH_2), 2.62 (4H, m, CH_2), 1.25 (18 H, s, ^{t}Bu), 1.18 (6H, s, CH_2-C-CH_3). 0.97 (6H, s, CH_2-C-CH_3); ^{13}C APT NMR (125 MHz, $CDCl_3$) δ/ppm = 174.7, 159.5 (br), 139.5 (br), 128.0, 121.9, 63.1, 55.6, 33.6, 31.5, 28.1, 21.1, 20.8. Elemental analysis: calculated C 41,86, H 5,03, N 5,28, found C 42.13, H 5.59, N 5.36. FTMS + p ESI (m_z, g/mol): found 2789.63, 1809.44, 829.24, 741.30, 681.28, 513.25 and 339.14; FTMS - p ESI (m_z, g/mol): found 2107.30, 1129.12, 510.78, 380.83 and 315.93.

Synthesis of $[LZn_2](p\text{-}TSO_3)_2$ (2)

A solution of H_2L (412 mg, 0.745 mmol) in dry THF (10 mL) was cooled to 0 °C under argon atmosphere. NaH (52.1 mg, 2.17 mmol) was added slowly. The solution was warmed to room temperature and left stirring for 1 hour. Zinc(II) *bis*-(*para*-toluene sulphonate) (479 mg, 1.548 mmol) was then added to the solution and the mixture was heated to reflux for 2 hours. A white precipitate was filtered off, washed with THF and dried. The solid was extracted with CH_2Cl_2. The volatiles were removed from the filtrate in a partial vacuum to yield the product (67%).

$C_{49}H_{70}Cl_2N_4O_8S_2Zn_2$ (1105.26 g/mol); ^{1}H NMR (400 MHz, $CDCl_3$) δ/ppm = 7.67 (2H, br s, Ar-H), 6.80 (10H, s, Ar-H), 4.61 (4H, br s, NH), 4.08 (4H, d, CH_2), 2.84 (6H, m, CH_2), 2.44 (6H, m, CH_2), 2.19 (6H, s, CH_3, 1.28 (18H, s, t-Bu), 1.02 (6H, s, CH_2-C-CH_3), 0.89 (6H, s, CH_2-C-CH_3); ^{13}C APT NMR (125 MHz, $CDCl_3$) δ/ppm = 174.7, 139.5 (br), 128.5, 127.7, 123.0, 62.9, 55.9, 33.7, 31.7, 28.3, 21.3, 21.1; Elemental analysis: calculated C 53,22, H 6,11, N 5,07, found C 54.17, H 6.90, N 4.89. FTMS + p ESI (m_z, g/mol): 1875.59, 853.29, 780.22; FTMS - p ESI (m_z, g/mol): found 2217.59, 1195.30.

Copolymerisation of CO_2 and cyclohexene oxide

General procedure

The copolymerisation of CO_2 and cyclohexene oxide (CHO) were performed in a 160 ml stainless steel autoclave (Parr) equipped with a gas entrainment stirrer (700 rpm), cooling loop and high pressure ATR-IR probe. Later was connected with an optical fibre to a MATRIX-MF IR spectrometer (Bruker). Catalyst **1** or **2** (0.031 mmol) and, if applicable, α,ω-dihydroxy polypropylene oxide (MW 725 g/mol, d = 1.449 g/mL, 1.5 mL, 2.17 g, 3.0 mmol) was dissolved in CHO (15.0 mL, 148 mmol) and degased three times with CO_2 at room temperature. The autoclave was pressurised with CO_2 (20 bar), heated rapidly to 100 °C and the reaction monitored with IR spectroscopy with a scan rate of 1 scan every 5 minutes. After 20 hours the reaction mixture was cooled to 15 °C and the remaining pressure released slowly. The autoclave was opened and a sample taken for the NMR analysis. The remaining polymer was dissolved in CH_2Cl_2 and transferred to a 250 mL round-bottomed flask. The volatiles were removed in a partial vacuum at 50 °C.

Copolymerisation of CO_2 and CHO using $[LZn_2](CF_3SO_3)_2$ (1)

The copolymerisation of CO_2 and CHO with catalyst **1** (30.1 mg, 0.031 mmol) according to the general procedure afforded a colourless viscous oil. After removal of all volatiles, 88% of polymer was obtained.

^{1}H NMR (400 MHz, $CDCl_3$) δ/ppm = 4.59 (brm, -[(C$\underline{H}$)$_2$-O-C(O)-O-(C$\underline{H}$)$_2$]$_m$-), 4.21 (brm, -C$\underline{H}$-O-C(O)-O-X, X = end groups), 3.35 (brm, -C$\underline{H}$-O-C(O)-O-P, P = polymer chain), 2.00-1.17 (m, 8H, -[C$\underline{H_2}$]$_m$-) ^{13}C APT NMR (125 MHz, $CDCl_3$) δ/ppm = 153.2, 83.3, 71.7, 32.2, 29.5, 27.9, 23.0, 19.3; Elemental analysis: calculated C 71.66, H 9.98 % for 30 repetition units (3084/(0.049*142.15+0.951*98.07)=30) of polyethercarbonate, OH as terminal groups); found C 69.95, H 9.80; M_n 3082 g/mol, PDI 1.64.

Copolymerisation of CO_2 and CHO using $[LZn_2]$(*p*-$TSO_3)_2$ (2)

The copolymerisation of CO_2 and CHO with catalyst **2** (31.3 mg, 0.031 mmol) according to the general procedure afforded a colourless viscous oil. After evaporating all volatiles, 74% of polymer was obtained.

^{1}H NMR (400 MHz, $CDCl_3$) δ/ppm = 4.59 (brm, -[(C$\underline{H}$)$_2$-O-C(O)-O-(C$\underline{H}$)$_2$]$_m$-), 4.21 (brm, -C$\underline{H}$-O-C(O)-O-X, X = end groups), 3.35 (brm, -C$\underline{H}$-O-C(O)-O-P, P = polymer chain), 2.00-1.17 (m, 8H, -[C$\underline{H_2}$]$_m$-) ^{13}C APT NMR (125 MHz, $CDCl_3$) δ/ppm = 153.2, 83.3, 71.7, 32.2, 29.5, 27.9, 23.0, 19.3; Elemental analysis: calculated C 59.27, H 7.24 % for 19 repetition units (2735/142.15=19) of strictly alternating polycarbonate, OH as terminal groups); found C 59.39, H 7.11; M_n 2735 g/mol, PDI 1.33.

Copolymerisation of CO_2 and CHO using $[LZn_2](CF_3SO_3)_2$ (1) in the presence of α,ω-dihydroxy polypropylene oxide

The copolymerisation of CO_2 and CHO with catalyst **1** (30.1 mg, 0.031 mmol) in the presence of α,ω-dihydroxy polypropylene oxide (725 g/mol, 1.5 mL, 3 mmol) according to the general procedure afforded a colourless viscous oil. After evaporating all volatiles, 72% of polymer was obtained.

^{1}H NMR (400 MHz, $CDCl_3$) δ/ppm = 4.26 (brm, $-[(C\underline{H})_2$-O-C(O)-O-$(C\underline{H})_2]_m$-), 4.21 (brm, -C$\underline{H}$-O-C(O)-O-X, X = end groups), 3.69 (brm, -C$\underline{H}$-O-C(O)-O-P, P = polymer chain), 3.16 (O-C$\underline{H}$(CH_3)-C$\underline{H_2}$-O, starter) 1.77-1.10 (m, -[C$\underline{H_2}]_m$-, O-CH($\underline{CH_3}$)-) ^{13}C APT NMR (125 MHz, $CDCl_3$) δ/ppm = 153.8, 82.8, 71.6, 32.2, 30.2, 29.3, 27.8, 24.1, 22.8, 19.3,16.9; Elemental analysis: calculated C 69.68, H 10.05 % for 28 repetition units ((3567-725)/(0.049*142.15+0.951*98.07)=28) of polyethercarbonate, OH as terminal groups and PPG 750 as starter; found C 69.95, H 9.80; M_n 3567 g/mol, PDI 1.75.

Copolymerisation of CO_2 and CHO using $[LZn_2]$(*p*-$TSO_3)_2$ (2) in the presence of α,ω-dihydroxy polypropylene oxide

The copolymerisation of CO_2 and CHO with catalyst **2** (31.27 mg, 0.031 mmol) in the presence of α,ω-dihydroxy polypropylene oxide (725 g/mol, 1.5 mL, 3.0 mmol) according to the general procedure afforded a colourless viscous oil. After evaporating all volatiles, 72% of polymer was obtained.

^{1}H NMR (400 MHz, $CDCl_3$) δ/ppm = 4.26 (brm, $-[(C\underline{H})_2$-O-C(O)-O-$(C\underline{H})_2]_m$-), 4.21 (brm, -C$\underline{H}$-O-C(O)-O-X, X = end groups), 3.69 (brm, -C$\underline{H}$-O-C(O)-O-P, P = polymer chain), 3.16 (O-C$\underline{H}$(CH_3)-C$\underline{H_2}$-O, starter) 1.77-1.10 (m, -[C$\underline{H_2}]_m$-, O-CH($\underline{CH_3}$)-) ^{13}C APT NMR (125 MHz, $CDCl_3$) δ/ppm = 153.8, 82.8, 71.6, 32.2, 30.2, 29.3, 27.8, 24.1, 22.8, 19.3,16.9; Elemental analysis: calculated C 61.01, H 8.61 for 9 repetition units

of strictly alternating polycarbonate, OH as terminal groups and PPG 725 as starter); found C 61.19, H 8.64; M_n 2019 g/mol, PDI 1.24.

Spectra and additional material on catalyst 1

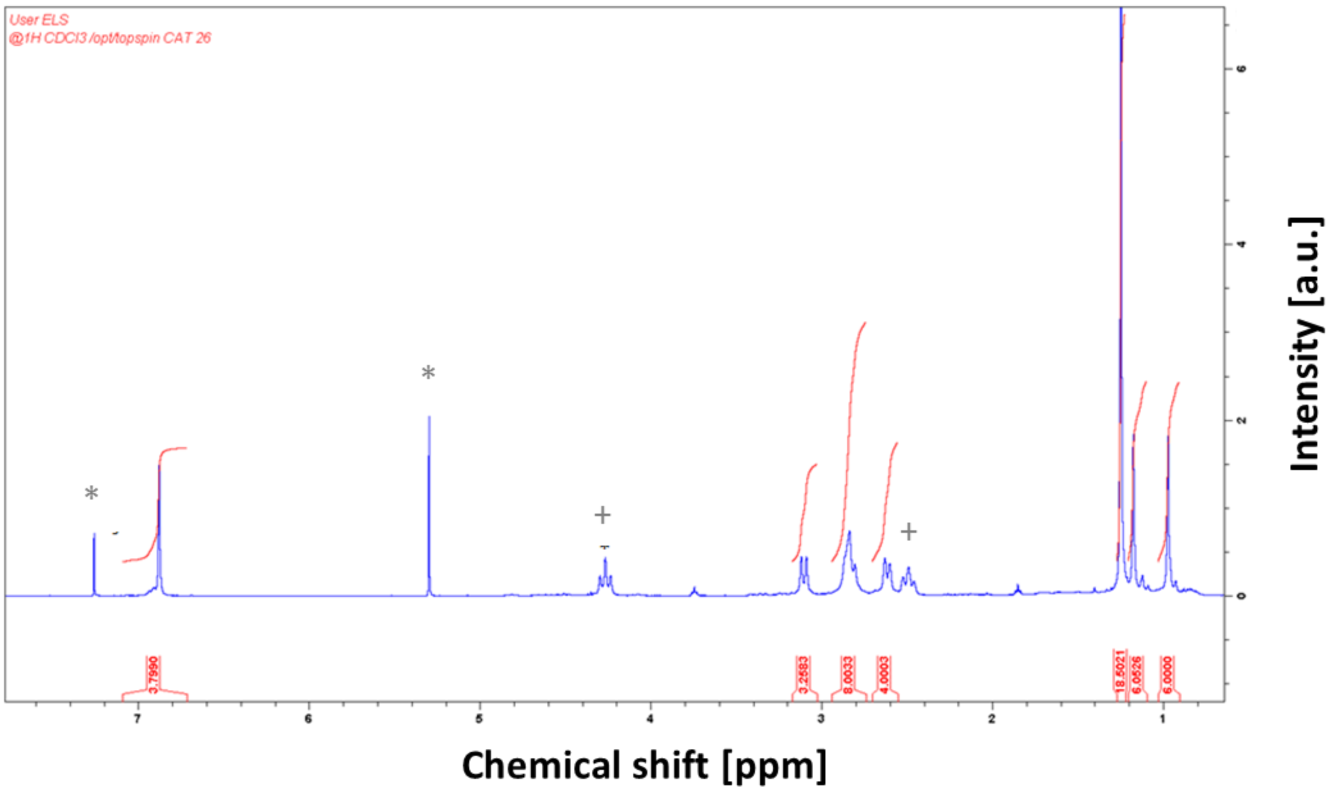

Figure S1. ^{1}H NMR spectrum of $[LZn_2](CF_3SO_3)_2$ (**1**) in $CDCl_3$ (* solvent signals; $^+$ thf [3]).

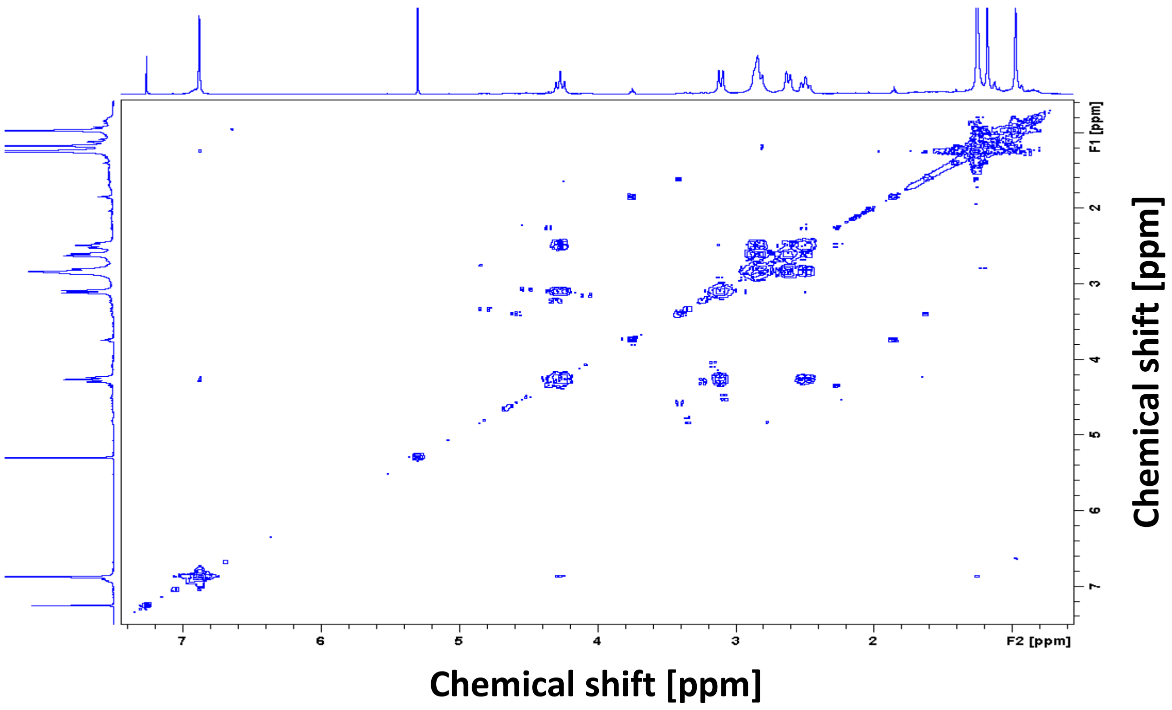

Figure S2. $^{1}H,^{1}H$-COSY NMR spectrum of $[LZn_2](CF_3SO_3)_2$ (**1**) in $CDCl_3$.

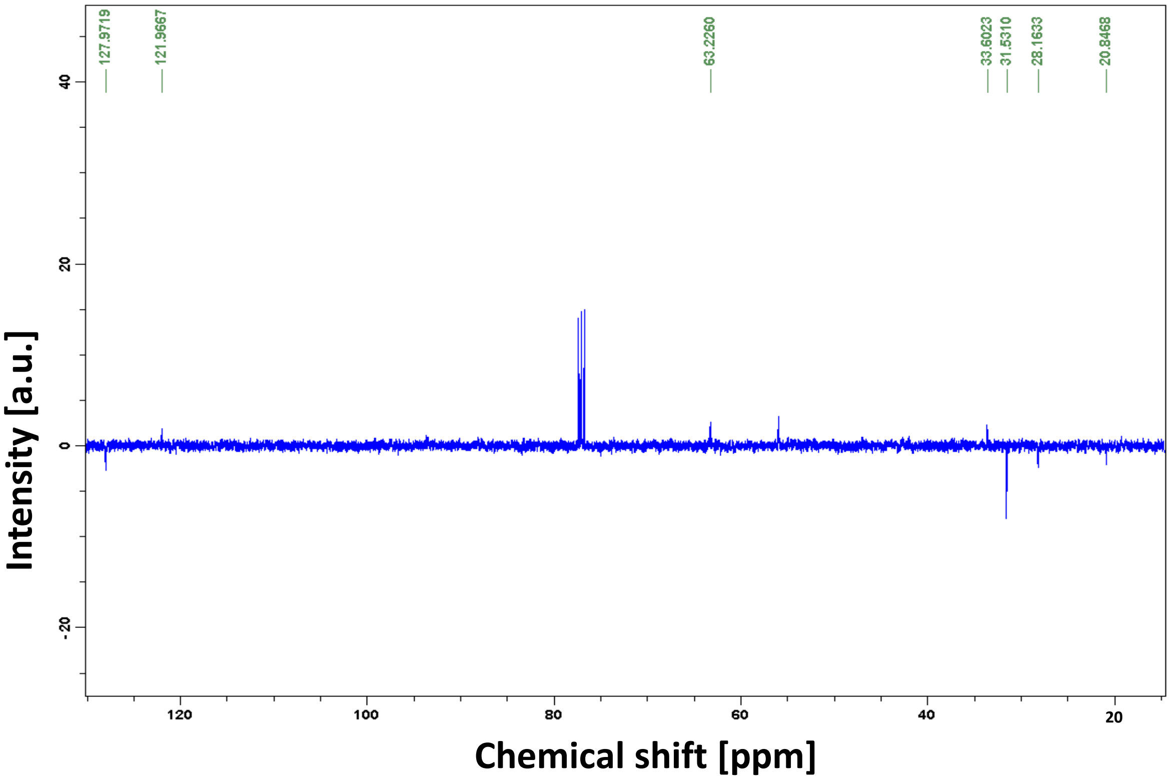

Figure S3. ^{13}C APT NMR spectrum of $[LZn_2](CF_3SO_3)_2$ (**1**) in $CDCl_3$.

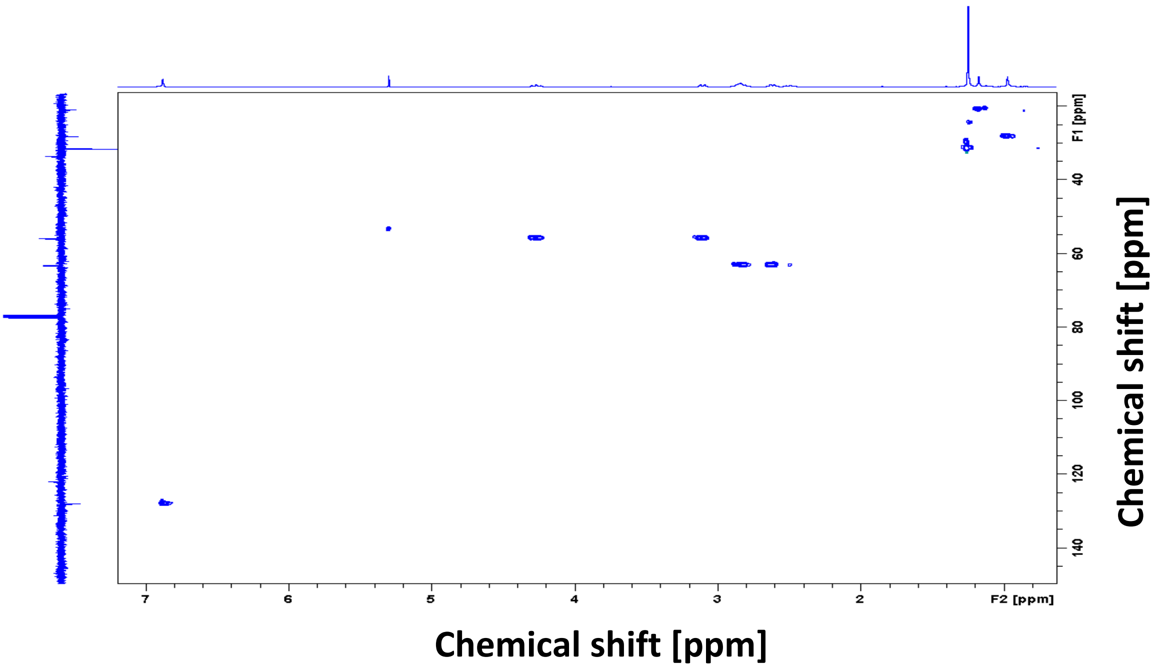

Figure S4. HSQC NMR spectrum of $[LZn_2](CF_3SO_3)_2$ (**1**) in $CDCl_3$.

Spectra and additional material on catalyst 2

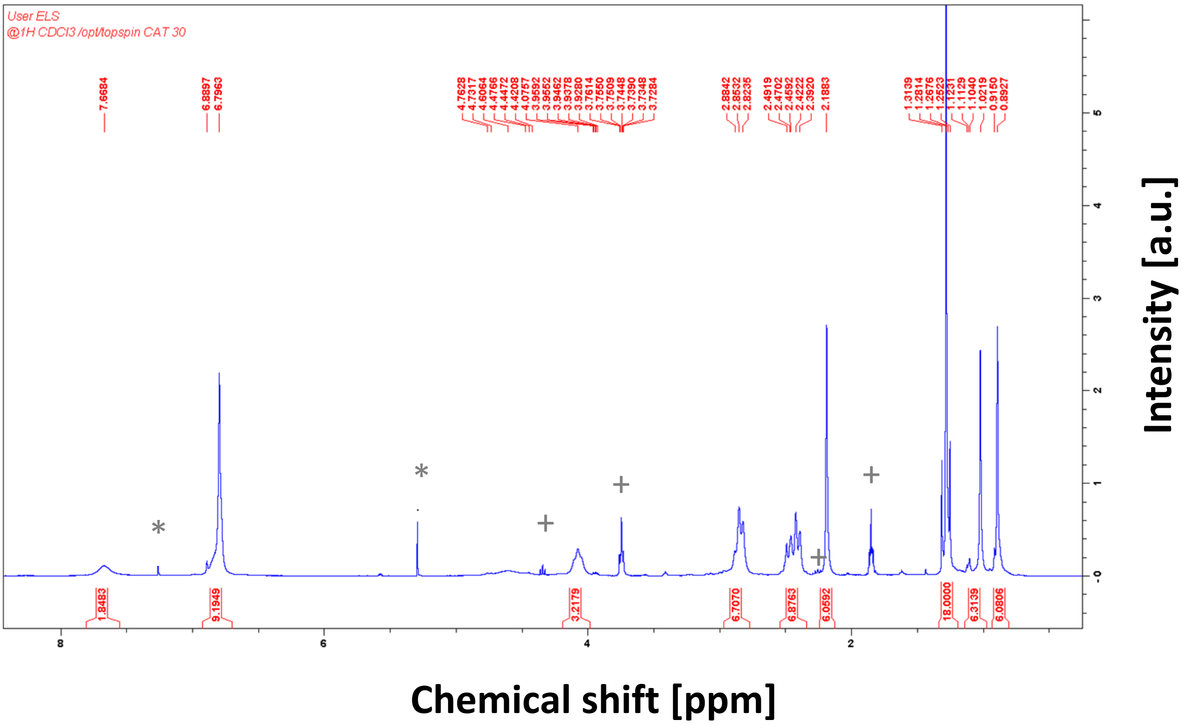

Figure S5. ^{1}H NMR spectrum of $[LZn_2](p\text{-}TSO_3)_2$ (**2**) in $CDCl_3$ (* solvent signals; $^{+}$ signals assigned to coordinated and free thf [3].

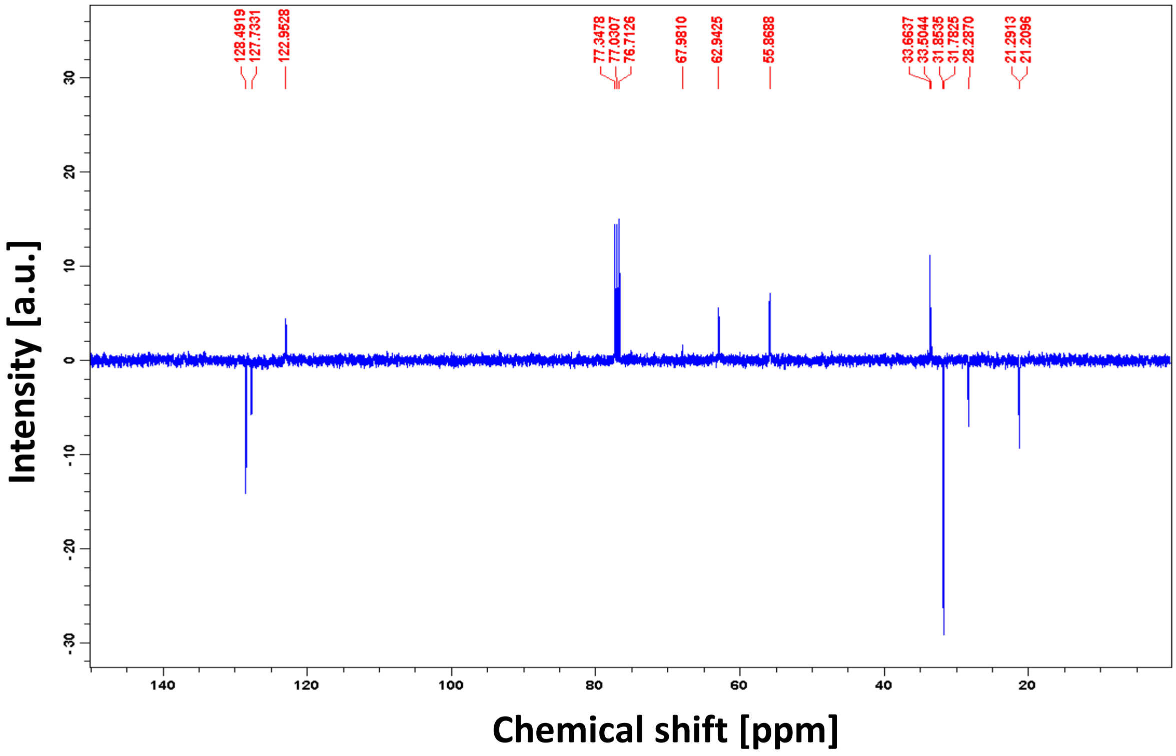

Figure S6. ^{13}C APT NMR spectrum of $[LZn_2](p\text{-}TSO_3)_2$ (**2**) in $CDCl_3$.

Comparison and assignment of the NMR signals

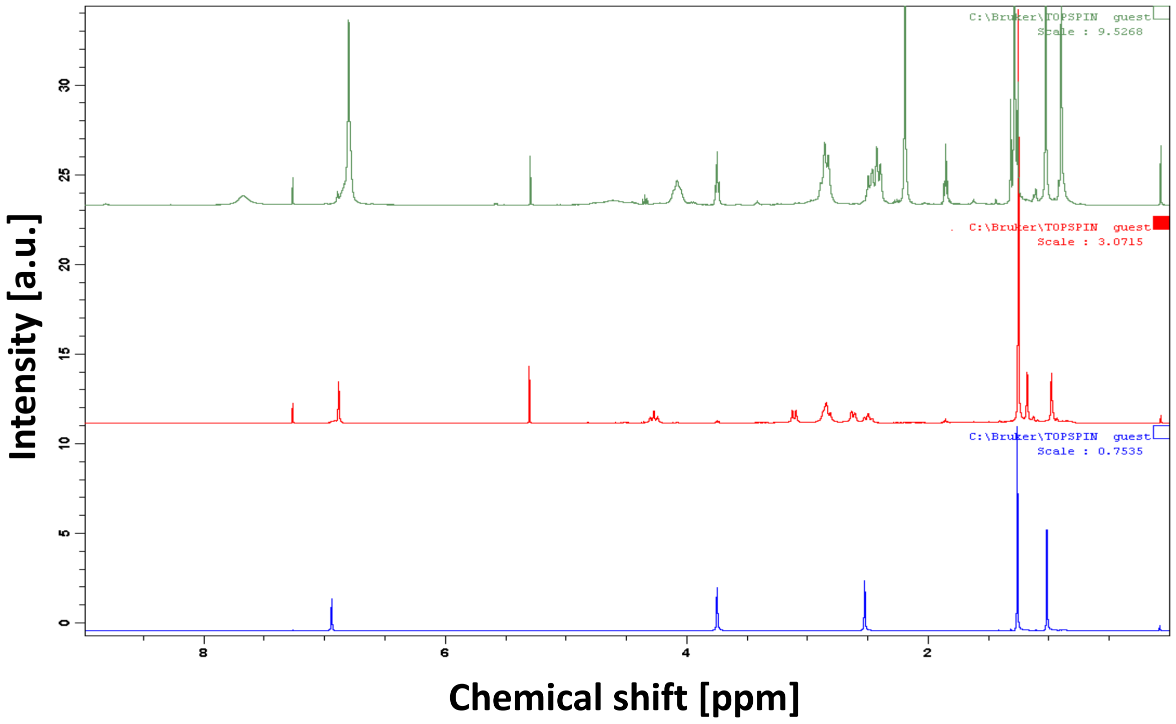

Figure S7. ^{1}H NMR spectra of $[LZn_2](p\text{-}TSO_3)_2$ (**2**, green, top), $[LZn_2](CF_3CO_2)_2$ (**1**, red, middle) and H_2L (blue, bottom) in $CDCl_3$.

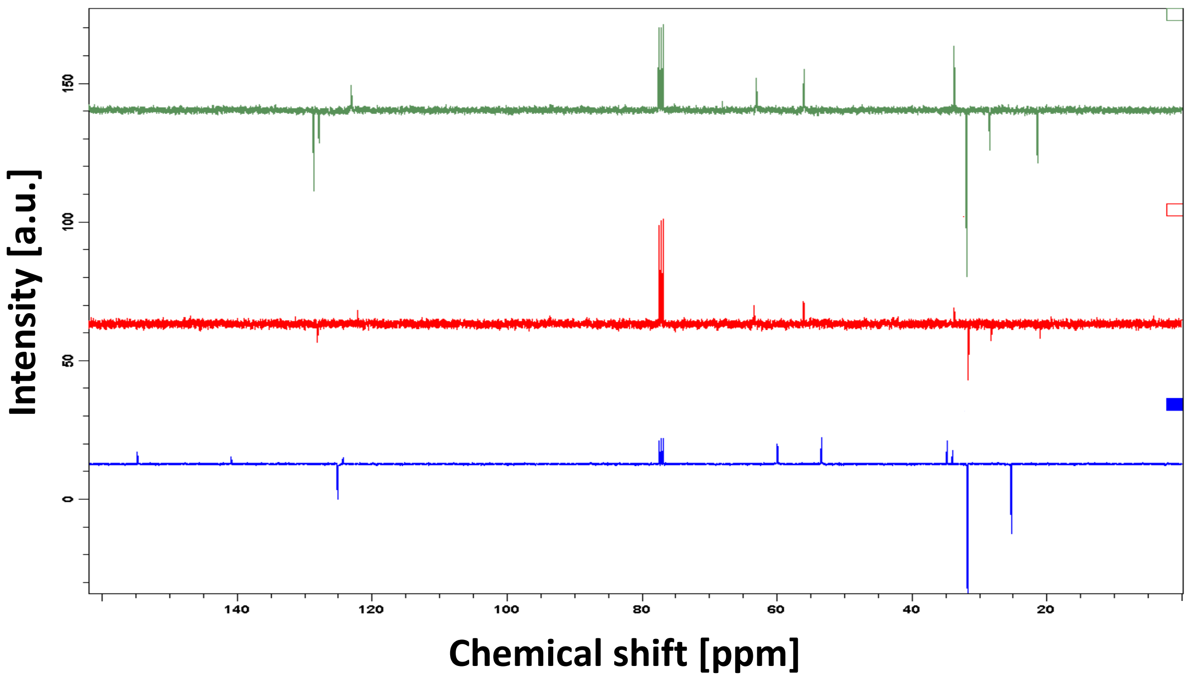

Figure S8. ^{13}C APT NMR spectra of $[LZn_2](p\text{-}TSO_3)_2$ (**2**, green, top), $[LZn_2](CF_3CO_2)_2$ (**1**, red, middle) and H_2L (blue, bottom) in $CDCl_3$.

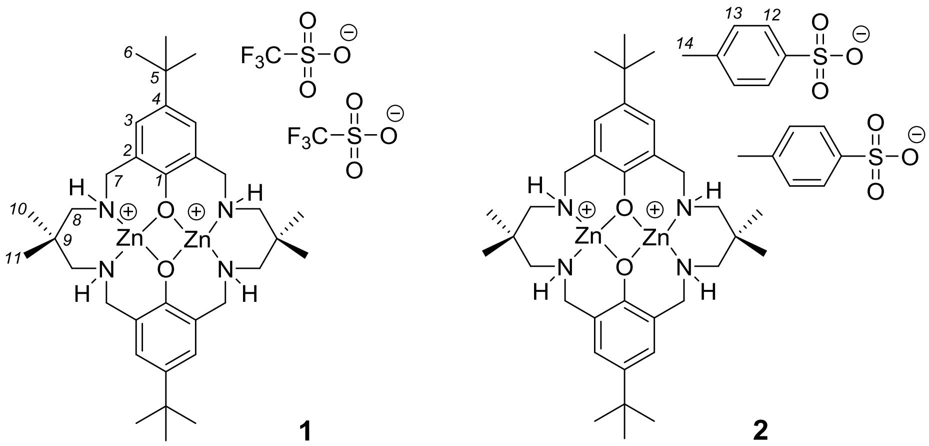

1 **2**

Scheme S1 Assignment of the NMR signals to the structure of the binuclear zinc complexes **1** and **2** employed in this study

Additional information concerning the copolymerisation of CO_2 and CHO using the complexes $[LZn_2](CF_3SO_3)_2$ (1) and $[LZn_2](p\text{-}TSO_3)_2$ (2) as catalyst

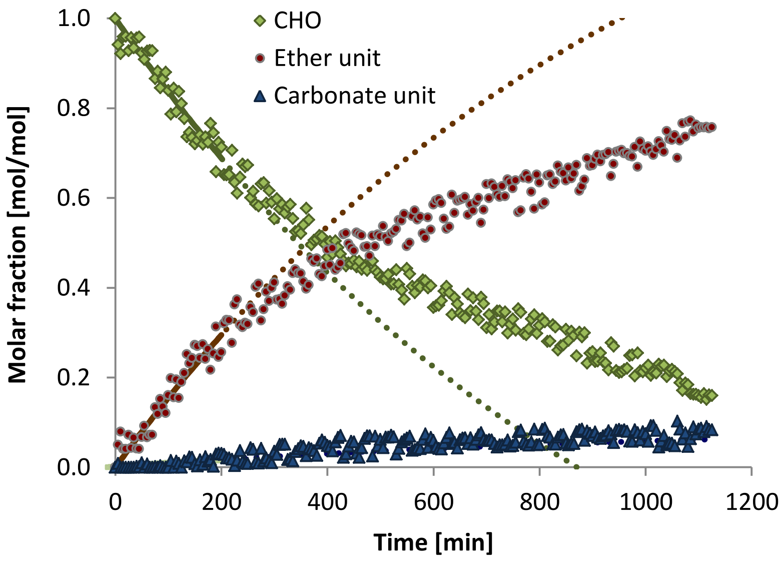

Figure S9. Time-concentration profile of the copolymerisation of CO_2 and CHO in the presence of catalytic amounts of complex **1** and fit of the initial period (0 – 200 min) according to a profile first order in CO_2 and zero order in epoxide extrapolating to the overall reaction time.

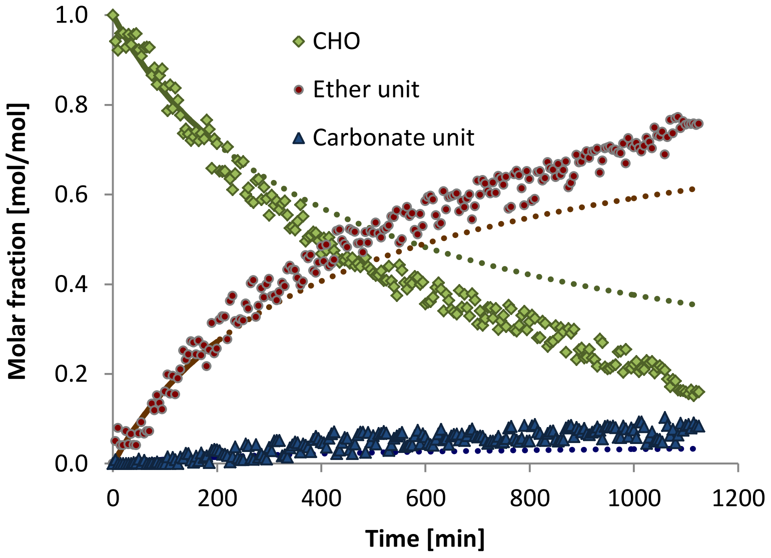

Figure S10. Time-concentration profile of the copolymerisation of CO_2 and CHO in the presence of catalytic amounts of complex **1** and fit of the initial period (0 – 200 min) according to a profile first order in CO_2 and second order in epoxide extrapolating to the overall reaction time.

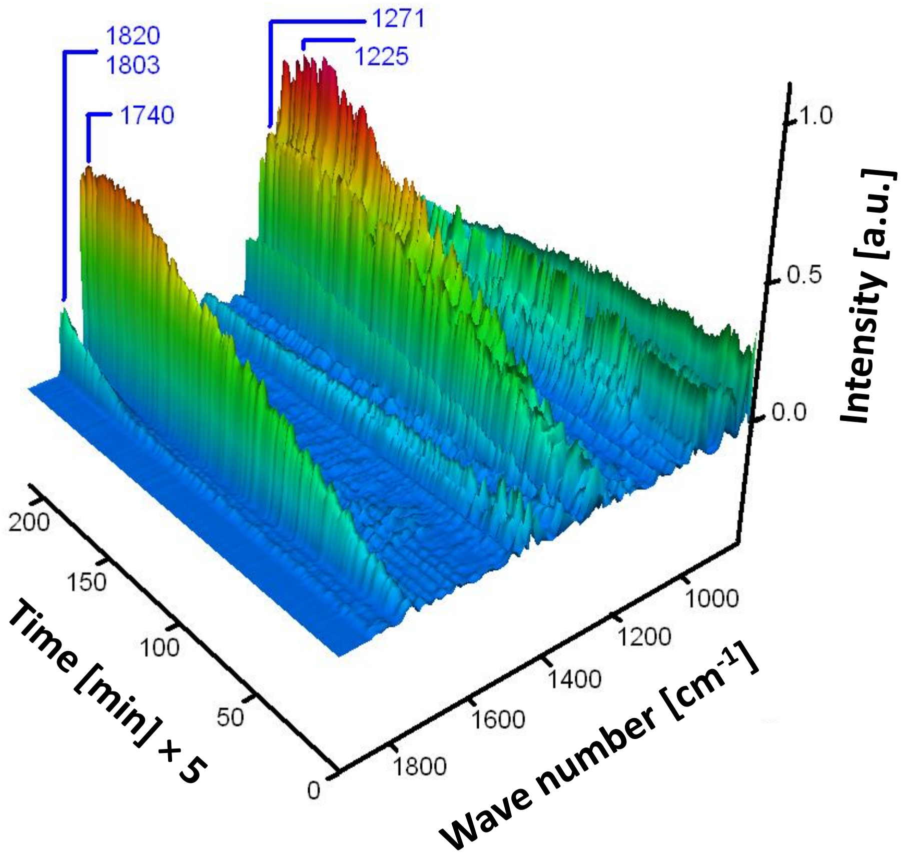

Figure S11. Time-resolved IR spectra of the copolymerisation of cyclohexene and CO_2 with catalyst **2**.

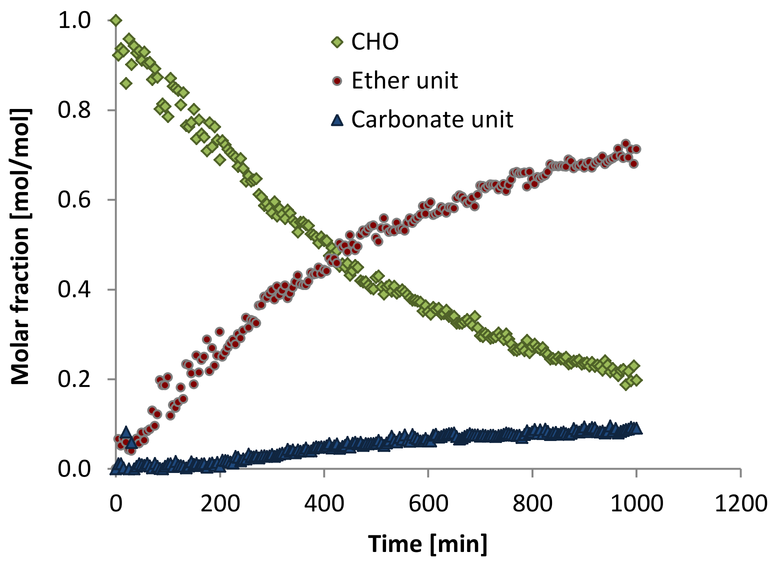

Figure S12. Time-concentration profile of the copolymerisation of CO_2 and CHO in the presence of α,ω-dihydroxy polypropylene oxide and catalytic amounts of complex **1**.

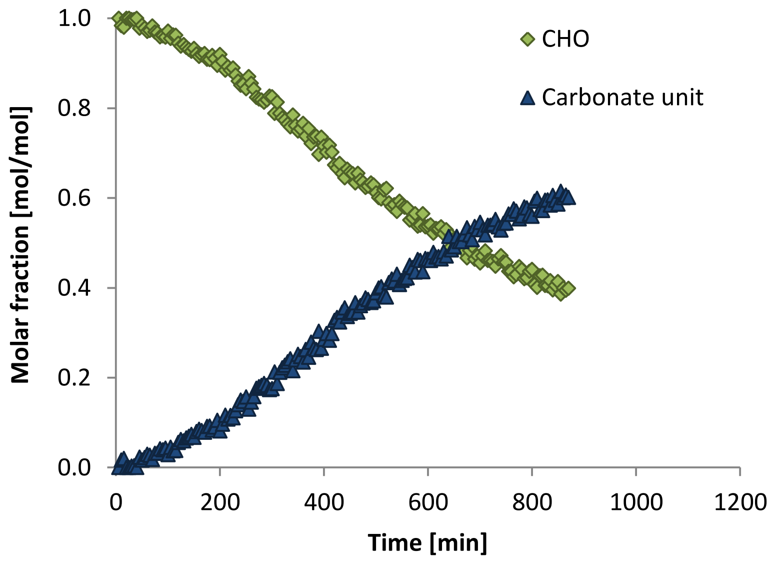

Figure S13. Time-concentration profile of the copolymerisation of CO_2 and CHO in the presence of α,ω-dihydroxy polypropylene oxide and catalytic amounts of complex **2**.

References

1 Kember; M. R.; Buchard, A.; Williams, C. K. *Chem. Commun*. **2011**, *47(1)*, 141-163.

2 Lehenmeier, M. W.; Bruckmeier, C.; Klaus, S.; Dengler, J. E.; Deglmann, P.; Ott, A.-K.; Rieger, B. *Chem. Eur. J*. **2011**, *17(32)*, 8858-8869.

3 Fulmer, G. R.; Miller, A. J.; Sherden, N. H.; Gottlieb, H. E.; Nudelmann, A.; Stoltz, B. M.; Bercaw, J. E.; Goldberg, K. I. *Organomet*. **2010**, *29*, 2176-2179.
